# Examining identical twins undergoing bariatric surgery: the single anastomosis duodeno–ileal bypass with sleeve gastrectomy (SADI-S) approach

**DOI:** 10.1093/jscr/rjae192

**Published:** 2024-03-27

**Authors:** Rahul Menon, Philip Lockie

**Affiliations:** General Surgery Department, Ipswich Hospital, Chelmsford Ave, Ipswich, QLD 4305, Australia; General Surgery Department, Ipswich Hospital, Chelmsford Ave, Ipswich, QLD 4305, Australia

**Keywords:** bariatric surgery, morbid obesity, monozygotic twins, laparoscopic surgery

## Abstract

Studies in monozygotic (MZ) twins may help researchers elucidate the complex relationships between genetic and environmental factors on weight loss. We present a world first of MZ twins who have undergone the single anastomosis duodenal-ileal bypass with sleeve gastrectomy (SADI-S) procedure who have identical weights 3 years post-operatively. Two MZ twin 49-year-old females were assessed preoperatively and were indicated for the SADI-S procedure. They underwent surgery in 2020 by the same surgical team. Three years later post-operatively they had identical weights of 62 kg (and a BMI of 23) and %EWL of 126 and 124% respectively. SADI-S is a novel bariatric procedure for morbid obesity. Studies have found concordant epigenetic patterns in patients who have undergone bariatric surgery as well as MZ twins who have hypocaloric diets. Genetics exert a strong influence in weight management. Surgical management as well as a collaborative multidisciplinary approach is beneficial in supporting long lasting weight loss in bariatric surgery.

## Introduction

In 2022, almost two thirds (65.8%) of adults in Australia were overweight or obese [1]. Over the last decade, the proportion of obese adults has increased and those with severe obesity are sitting at 13% [1]. When considering the effective management of long-term weight loss, lifestyle interventions, pharmacological interventions, and behavioural mechanisms are needed.

To further elucidate genetic as well as environmental influences on weight loss after bariatric surgery, we present the world’s first experience with monozygotic (MZ) twins using the single anastomosis duodenal-ileal bypass with sleeve gastrectomy (SADI-S) approach.

### Case report

Two MZ twin females aged 49 were evaluated preoperatively by an identical multidisciplinary bariatric surgery team. Both patients had endoscopic placement of an intra-gastric balloon with minimal resultant weight loss. Preoperative weight for twin 1 was 93.2 kg (with a BMI of 35.1 kg/m^2^) and twin 2 was 89 kg (with a BMI of 33.1 kg/m^2^). Both twins had normal blood tests and no past medical history of note. The operation for both twins was completed a few weeks apart. In both patients, the abdomen was entered with a standard 12 mm port. Other ports were inserted under vision. The stomach was mobilized along the greater curvature with an energy device Harmonic Ace (Ethicon, Johnson&Johnson, NJ, USA). Sleeve gastrectomy was performed over a 38 Fr bougie and a 60 mm powered stapler device Echelon (Ethicon, Johnson&Johnson, NJ, USA). Resected stomach was removed and small bowel retrograde was measured at 300 cm for both patients. A loop duodeno-ileal anastomosis was created and the patient was closed in the normal fashion.

No complications were observed in either patient, and they were discharged in 48 h without any problems. The patients received regular nutritional, dietetic and psychological support throughout the post-operative period. At 3 years, both patients had identical weight of 62 kg (BMI of 23 kg/m^2^) and %EWL of 126 and 124%, respectively.

## Discussion

We present the first report examining the changes in BMI and %EWL in MZ twins after a SADI-S procedure. At 3 years post-surgery, we observed several factors including psychological and social support as well as the importance of diet post-bariatric surgery. It is important to understand how complex interplays between genetics have a strong influence of weight management, which is supported by the extant literature [2, 3].

The SADI-S is a novel and technically advanced hypo-absorptive bariatric procedures indicated for Class III obesity. Initially biliary diversion procedures were proposed by DeMeester et al., in 1987 as a suitable method for the duodenal inhibition of biliary reflux [4]. Other procedures only reduced symptoms partially and combined a significant complication rate [4–6]. Instead, a biliary diversion procedure with a single intestinal anastomosis was developed to prevent biliary reflux but also to prevent the potentially long-term and severe side effects associated [4].

Retrospective Study data have demonstrated that SADI-S has resulted in significant weight loss with a concurrent improvement in metabolic conditions like diabetes, dyslipidaemia, and obstructive sleep apnoea (OSA) [7–9]. A meta-analysis of 581 patients by Shoar et al. suggested an average excess weight loss percentage of 80% at 2 years [10]. Co-morbidity resolution rate was 74.1% for type 2 diabetes mellitus, 96.3% for hypertension, 68.3% for dyslipidaemia, 63.3% for OSA, and 87.5% for GORD [10]. SADI-S can be used both as a primary procedure, but also as a revision procedure when other bariatric procedures have failed or to stage subsequent procedures.

Genetic homozygosity in MZ twins helps us examine the complex interplay between genetic and environmental factors in the management of weight post-bariatric surgery. There is widespread controversy on the strength of genetic contribution to obesity and some researchers claim that they have a greater influence than environmental influences [11–13]. Concordance rates for degrees of obesity are twice as high for MZ twins as for dizygotic twins [14]. Adoption studies have examined genetic resemblance further with adoptees resembling the BMI of their biological parents and siblings but not of their adopted parents [15]. Stunkard et al. further compare the twins reared apart and together noting the intrapair correlation coefficients of twins reared apart closely resemble those of twins who are reared together [15]. These findings further corroborate earlier studies and suggest that genetic influences of BMI are substantial with environmental factors being less significant [15].

Epigenetic patterns in patients with obesity or concurrent metabolic disease have been observed in DNA analysis or blood samples from individuals with severe obesity [16]. Talukdar *et al.* have identified 41 significant epigenetic targets associated with weight loss due to bariatric surgery of which were also partially replicated in BMI-discordant MZ twins who followed a hypocaloric diet [16]. This suggests that bariatric surgery-induced weight loss improves both clinical and biochemical markers in health that are similarly seen in MZ twin studies regardless of the methods of weight loss [16]. Our own case study shows similar %EWL as well as similar improvements in metabolic markers suggesting that similar epigenetic changes could be observed in this cohort.

Morbidly obese patients are also afflicted with psychological comorbidities [17] and psychological support is integral in any bariatric programme. Frequent users of an online application based social support group post-bariatric surgery showed higher weight loss up to 2 years than compared with non-users [18], a meta-analysis examining this supports this further but boasts more modest results, with weight loss being similar at 6 months but being greater and remaining significant at 12 months [17]. Social support is an ideal platform to provide consistent psychological, emotional, and nutritional support throughout a bariatric programme and can promote a healthy lifestyle and prevent old habits from resurfacing [17].

## Conclusion

The extant literature suggests that genetics exert a strong influence of obesity and the management of weight and metabolic conditions. Bariatric surgery in the morbidly obese remains the most effective method of lasting weight loss but post-operative social and psychological support are significant factors. MZ twins are ideal subjects to study genetic and environmental influences on weight loss, but further research will help identify which factors exert a more significant influence.

## Author contributions

Literature review and drafting the manuscript: Rahul Menon. Philip Lockie provided guidance pertaining to the design and scope of the study. All authors have read and agreed to the published version of the manuscript.

## Conflict of interest statement

None declared.

## Funding

None declared.

## Ethical approval

All procedures performed in studies involving human participants were in accordance with the ethical standards of the institutional and/or national research committee and with the 1964 Helsinki Declaration and its later amendments or comparable ethical standards.

## Informed consent

For all participants described in this study, full informed consent was sought.
